# Effect of lithium on circadian activity level and flexibility in patients with bipolar disorder: results from the Oxford Lithium Trial

**DOI:** 10.1016/j.ebiom.2025.105676

**Published:** 2025-04-02

**Authors:** Ni Xu, Yan Yan, Kate E.A. Saunders, John R. Geddes, Michael Browning

**Affiliations:** a Department of Psychiatry, University of Oxford, Oxford, United Kingdom; b Peking University Sixth Hospital, Beijing, China; c Peking University Institute of Mental Health, Beijing, China; d NHC Key Laboratory of Mental Health (Peking University), Beijing, China; e National Clinical Research Center for Mental Disorders (Peking University Sixth Hospital), Beijing, China; f Department of Psychology, Stanford University, Stanford, California, USA; g Oxford Health NHS Trust, Oxford, United Kingdom

**Keywords:** Bipolar disorder, Lithium, Circadian rhythm, Actigraphy, Experimental medicine

## Abstract

**Background:**

Disruption of circadian rest-activity is prevalent in patients with bipolar disorder (BD). Lithium's impact on circadian rhythms has been documented in cell lines, animal models, and pharmacogenomics studies in patients with BD. However, the causal relationship between such disruption and BD remains unclear.

**Methods:**

We investigated the early effects of lithium on circadian rest-activity in an exploratory analysis of a randomised, placebo-controlled, double-blind six-week study on patients with BD. Participants were assigned to receive either lithium or a placebo in a 1:1 ratio. Circadian activity was monitored using actigraphy, and daily affect was assessed through ecological momentary assessment. A computational model was used to quantify different types of activity variability, and the impact of lithium on activity level, activity onset time and their variability were analysed using linear mixed models.

**Findings:**

Of the thirty-five participants who began treatment, 19 received lithium and 16 received a placebo. Lithium significantly altered circadian rest-activity patterns, including reducing daytime activity levels (after 4 weeks, below as well: Cohen's d = −0.19, p = 0.002, linear mixed model, ibid.), advancing the onset of daytime activity (Cohen's d = −0.14, p = 0.018), and increasing the volatility of both daytime activity level (Cohen's d = 0.10, p = 0.002) and its onset time (Cohen's d = 0.13, p < 0.001), independent of affective symptoms changes.

**Interpretation:**

This study establishes a causal link between lithium treatment and reduced circadian activity with advanced circadian phase, potentially via temporarily increasing their volatility (flexibility). Significant circadian changes were detected within one week of starting lithium, highlighting their potential as an early biomarker for treatment response.

**Funding:**

This research was supported by the 10.13039/100010269Wellcome Trust Strategic Award (CONBRIO: Collaborative Oxford Network for Bipolar Research to Improve Outcomes, reference No. 102,616/Z), NIHR Oxford Health Biomedical Research Centre and the NIHR Oxford cognitive health Clinical Research Facility.

**Research in context**

**Evidence before this study**

Lithium's influence on circadian rhythms is well-documented across cell line experiments, animal studies, and pharmacogenomics research involving patients with bipolar disorder (BD). Despite this, the causal impact of lithium on circadian rhythms in patients with BD remains poorly defined. To address this gap, we performed a systematic review to determine whether lithium affects circadian rhythms in patients with BD. Our review encompassed both experimental and observational studies that primarily focused on circadian rhythm outcomes measured with validated, real-world applicable tools. We searched databases such as the Cochrane Library, EMBASE, MEDLINE, PsycINFO, and clinicaltrials.gov for both published and unpublished studies from their inception until September 17, 2020. Our search revealed no experimental studies with a low risk of bias. However, a meta-analysis of four observational studies indicated a potential association between lithium treatment and increased morningness in patients with BD. An updated search conducted on May 4, 2024 failed to identify any new experimental studies that clarify the causal relationship between lithium and circadian rhythms in patients with BD.

**Added value of this study**

This research represents a randomised, double-blind, placebo-controlled study to use high-frequency sampling of activity and affect combined with computational modelling to explore the causal relationship between lithium treatment and circadian rest-activity in patients with BD. Our findings indicate that lithium induces significant changes in circadian rest-activity as early as one week after initiation of treatment, independent of changes in affective symptoms. Specifically, lithium was found to reduce daytime activity levels and advance the onset time of daytime activity. Furthermore, we implemented a novel computational method, a Bayesian filter, to parse out different sources of activity variability. Our analyses revealed that lithium increased the volatility of both daytime activity and its onset time. This effect could be interpreted as a perturbative mechanism facilitating the transition of patients to a healthier circadian pattern.

**Implications of all the available evidence**

Our study underscores the critical role of circadian rest-activity in the psychopathology of BD and in understanding lithium's mechanism of action. By identifying early and mood-independent alterations in circadian rest-activity after lithium treatment, our research not only enhances our understanding of BD but also aids in the development of early biomarkers for the therapeutic effects of lithium. The stabilisation of circadian rhythms following lithium treatment could serve as an early marker of its efficacy, potentially guiding the personalised selection of mood stabilisers. To establish a connection between early-stage changes and long-term clinical outcomes, future research will need to include long-term follow-up studies. Additionally, our findings hint at a mechanism by which the mood-stabilising effect of lithium may be mediated through earlier behavioural changes. Confirming this will require mediation analysis in a larger sample to solidify these preliminary insights.

## Introduction

Bipolar disorder (BD) is a class of chronic mental disorders characterised by severe, episodic mood episodes and disturbed activity levels.^1^ Activity changes have gained recognition as a cardinal symptom of BD alongside mood changes.^2^ Notably, DSM-5 criteria now include increased activity as a core symptom of mania and hypomania, with studies suggesting that energy changes may be a better predictor of future mood episodes than mood changes.^3^

In addition to abnormal activity levels, circadian rest-activity disruption has also been observed in BD. Circadian rest-activity refers to the 24-h cycle of motor activity governed by the body's internal clock. Patients with BD often exhibit a less stable circadian rest-activity pattern,^4^ which is linked to greater mood variability, more manic symptoms, and delayed circadian phase.^5^^,^^6^ Additionally, patients with BD are more likely to have an evening chronotype and are at higher risk for circadian rhythm sleep-wake disorders, particularly delayed sleep-wake phase disorders.^4^^,^^7^ This circadian disruption is further evidenced by a significantly delayed onset of melatonin in BD compared to those with unipolar depression.^8^

Lithium has been recognised as the gold-standard treatment for BD.^9^ However, its mechanism of action is only partially understood. Interest in lithium's effects on circadian rhythms is growing, with evidence from studies in cell lines, animals, and pharmacogenomics suggesting that lithium can influence circadian rhythms.^10^^,^^11^ However, evidence specific to patients with BD is still limited. Our previous systematic review suggested a possible association between lithium use and greater morningness in patients with BD, however, the predominance of cross-sectional studies limited causal inferences.^12^ Therefore, experimental studies are needed to establish causality in this field.

Beyond circadian-rest activity and its onset time, the variability of these rhythms has garnered interest, offering insights into the biological clocks affected by lithium. A computational modelling approach has been developed to characterise mood instability in patients with BD, suggesting that mood instability can be decomposed into two different types of variability, volatility and noise.^13^ Volatility reflects the persistent shifts in mood over time such that a change in mood is more likely to sustain than dissipate, while noise represents the transient fluctuations in mood that do not last. Under this framework, observed mood at a certain time point is the combination of three latent variables: mean, volatility, and noise. Although these latent variables cannot be directly measured, computational methods allow their inference.

Applying this approach, Pulcu and colleagues estimated the volatility and noise of self-reported positive affect (PA) and negative affect (NA) in patients with BD, borderline personality disorder, and healthy controls.^14^ They reported that while both BD and borderline personality disorder were associated with increased mood instability, BD was distinctively characterised by increased affective volatility compared to borderline personality disorder. Importantly, lithium treatment for BD selectively increased the volatility of PA across two studies, indicating that its therapeutic effect may be mediated by increased flexibility in affective states.

The current study investigated the early effects of lithium on circadian rest-activity in patients with BD through a randomised, placebo-controlled, double-blind, six-week experimental study (Oxford Lithium Trial, Oxlith).^15^ The study examined lithium's impact on activity levels, onset times, and two types of variability.^14^ Echoing previous meta-analytic findings, we hypothesised that lithium would reduce daytime activity intensity and advance the daytime activity onset time (indicating a more morningness chronotype). Given previous findings of the effect of lithium on affective variability, we further hypothesised that lithium might decrease activity level and restore circadian rest-activity pattern via modifying their volatility levels.

## Methods

The Oxlith Lithium (Oxlith) trial is a randomised, placebo-controlled, double-blind, six-week experimental study of lithium treatment in patients with BD and mood instability.^15^ Its purpose is to evaluate early affective, cognitive, neural, and biochemical effects of lithium among patients with BD and current mood instability, while the effect on circadian rest-activity was an exploratory aim. The effect of lithium on affective variability has been reported previously.^14^

Participants were initially recruited from primary care and community mental health teams at Oxford. Each participant had been diagnosed with BD and had reported significant mood instability, but was not experiencing an acute episode that necessitated immediate treatment. Psychiatrists assessed patients' mood instability during the screening visit based on their history, past experiences, and mental status examination. The full inclusion criteria were:

1) Willing and able to consent to participate in the trial
2) Age 18 or over
3) Male or female (self-reported)
4) Diagnosis of BD (I, II, not otherwise specified) based on DSM-IV (patients with BD who have comorbid anxiety or borderline personality disorder are not excluded)
5) Clinical complaint of significant mood instability
6) Clinical uncertainty of benefit of lithium treatment
7) Not on lithium at trial entry
8) No clear indication for immediate treatment with lithium
9) No clear indication for alternative treatment
10) Pre-treatment renal, cardiac, thyroid, and parathyroid function testing is acceptable for lithium initiation
11) Willing and able to comply with all trial requirements over an eight-week period, as assessed by a psychiatrist

The full exclusion criteria were:

1) Any contraindication to lithium
2) Presenting a need for treatment of an acute mood episode where placebo would be inappropriate
3) Currently taking any psychotropic medication that cannot be withdrawn (e.g., antidepressants, antipsychotics, other mood stabilisers, benzodiazepine, non-benzodiazepine sleeping tablets)
4) Clinically significant substance abuse
5) Participation in another medication trial in the past 12 weeks
6) Immediate risk of suicide and self-harm
7) Pregnant, lactating or planning pregnancy over the next 12 weeks

Prior to randomisation, psychiatrists reviewed the frequency and intensity of their changes in mood symptoms, which had been monitored in the pre-randomisation phase to re-assess mood instability. In addition, psychiatrists assessed each participant's potential benefits from lithium treatment, taking into account the nature of their disorder and the required commitment to long-term lithium use. Participants for whom the benefits of lithium were uncertain, and therefore in whom the potential randomisation to receive placebo was acceptable, were enroled in the study.

For instance, an individual might have received their diagnosis recently or have had relatively few major mood episodes. All enroled participants discontinued psychotropic medications prior to the start of the study.

Participants who met the eligibility criteria entered the pre-randomisation phase, which lasted for approximately two weeks. The pre-randomisation phase provided a pre-treatment assessment of participants' activity level by wrist-worn actigraphy and familiarised them with the daily and weekly digital symptom monitoring tools. At the end of the pre-randomisation phase, each participant attended a randomisation visit when they were randomly allocated to either the lithium or placebo group. Lithium carbonate was provided as Priadel 200 mg prolonged-release tablets. Matched placebo tablets were manufactured by The Guy's and St Thomas NHS Foundation Trust Pharmacy Manufacturing Unit. Each bottle of Priadel and placebo contained 28 tablets and was assigned a unique randomisation number to ensure concealment of randomisation. Participants in the treatment group started lithium at 400 mg/day taken as a single dose at night. The target lithium serum level was 0.7 mmol/L (range from 0.4 to 1.0 mmol/L). The dose of lithium was adjusted to maintain the serum lithium levels within the therapeutic range by following a predetermined schedule (Table 1). For participants in the placebo group, an unblinded nurse gave the trial psychiatrist a sham lithium level considering the reported adherence, time since most recent dose and adverse events. Recommended sham lithium level is listed in Table 2, and the psychotropic medication participants were on before the trial is listed in Table 3.

**Table 1:** Lithium dose adjustment scheme.

Lithium level	Titration/dose adjustment
≤0.3 mmol/L	Increase dose to 800 mg/day
>0.3 and <0.6 mmol/L	Increase dose to 600 or 800 mg/day as appropriate
0.6–1.0 mmol/L	Continue current dose
≥1.0 mmol/L	Decrease dose by 200 mg/day or 400 mg/day as appropriate

**Table 2:** Recommended sham lithium serum levels for patients allocated to placebo group.

Current dose	Reported adherence	Level reported to trial psychiatrist (mmol/L)
2 tablets (400 mg/day)	More than 65%	0.4
	Between 50 and 65%	0.3
	Between 25 and 49%	0.2
	Less than 25%	0.1
3 tablets (600 mg/day)	More than 65%	0.6
	Between 50 and 65%	0.4
	Between 25 and 49%	0.2
	Less than 25%	0.1
4 tablets (800 mg/day)	More than 65%	0.8
	Between 50 and 65%	0.6
	Between 25 and 49%	0.4
	Less than 25%	0.2
5 tablets (1000 mg/day)	More than 65%	1.0
	Between 50 and 65%	0.7
	Between 25 and 49%	0.4
	Less than 25%	0.2

**Table 3:** Breakdown of psychotropic medications previously used by individual participants prior to entering the trial.

Patient ID	Psychotropic medication used before trial	Patient ID	Psychotropic medication used before trial
1	Sertraline 100 mg/d Citalopram 20 mg/d	19	None
2	None	20	Propranolol 40 mg/d Sertraline 50 mg/d
3	Quetiapine 200 mg/d∗ Citalopram 20 mg/d∗	21	None
4	Olanzapine 10 mg/d Aripiprazole 10 mg/d Valproate 800 mg/d	22	Citalopram 20 mg/d Sertraline 75 mg/d Zopiclone 3.75 mg/d∗ Quetiapine 75 mg/d∗
5	Fluoxetine 40 mg/d∗	23	Quetiapine 150 mg/d∗ Sertraline 100 mg/d Mirtazapine NA
6	Lamotrigine 150 mg/d∗ Lofepramine 70 mg/d	24	None
7	Fluoxetine 20 mg/d	25	Citalopram 20 mg/d Lamotrigine 100 mg/d∗ Dosulepin NA Sertraline 50 mg/d
8	Quetiapine 50 mg/d∗ Diazepam 5 mg/d	26	Citalopram 30 mg/d Olanzapine 5 mg/d
9	Sertraline 50 mg/d	27	Amitriptyline 20 mg/d
10	Citalopram 20 mg/d Sertraline 50 mg/d	28	Mirtazapine 30 mg/d
11	Aripiprazole 7.5 mg/d Fluoxetine NA Risperidone 4 mg/d Haloperidol NA Carbamazepine NA Quetiapine 200 mg/d Olanzapine 10 mg/d Lamotrigine NA Amisulpride NA	29	Fluoxetine 20 mg/d∗ Quetiapine 25 mg/d∗
12	None	30	Quetiapine 150 mg/d Sertraline NA Venlafaxine 225 mg/d
13	Olanzapine 10 mg/d∗ Clonazepam 1 mg/d Citalopram 40 mg/d Sertraline 150 mg/d Lorazepam 2 mg/d Zopiclone 7.5 mg/d	31	None
14	Sertraline NA Mirtazapine 30 mg/d Citalopram 20 mg/d	32	Quetiapine 50 mg/d∗
15	Mirtazapine 15 mg/d∗ Citalopram 20 mg/d	33	Sertraline 100 mg/d Lamotrigine 150 mg/d∗ Quetiapine 50 mg/d
16	None	34	Citalopram 20 mg/d∗ Promethazine 20 mg/d∗
17	Citalopram 20 mg/d Fluoxetine 40 mg/d	35	None
18	Bupropion NA Lamotrigine NA Quetiapine 400 mg/d		

All psychotropic medications were discontinued before the start of the trial. ‘NA’ indicates that data were not available. The asterisks indicate psychotropic medication that was discontinued within one month prior to trial entry. The dosage indicates the highest dose ever used by this patient.

During the six-week randomised phase (including titration phase), participants attended four assessment visits at day 4, day 8, during the fourth week (day 21–28) and the sixth week (day 35–42) post-randomisation. Lithium serum level, reported treatment adherence, and adverse events were checked at each visit. At the 6-week visit, the psychiatrist and the patient were unblinded, and the psychiatrist discussed future treatment options with patients. The study enrolment period spans from August 2015 to January 2018, with data collection completed by February 2018.

### Mood measure

Participants completed the Positive and Negative Affect Schedule, 10-item version (PANAS)^16^ online on an iPad once per day. The ten items measure participant's positive affect (PA) and negative affect (NA) over the day, including feeling afraid, nervous, upset, hostile, ashamed, active, determined, attentive, inspired, alert. Each item is rated on a five-point scale from never to always (1–5, ‘never’ corresponds to 1, and ‘always’ corresponds to 5).

### Circadian rest-activity measure

Raw circadian rest-activity data was collected using GENEActiv Original actigraphs (ActivInsights Ltd., UK) with a triaxial ± 8 g seismic acceleration sensor (1 g = 9.81 m/s^2^). The measurement frequency was 25 Hz and autocalibration method was used to minimise the calibration error.^17^ The levels and the timing of circadian rest-activity were derived based on non-parametric analysis of raw activity data (see Data preprocessing section below). Two types of variability, volatility and noise, were derived from the circadian rest-activity level and timing using the Bayesian filter.

#### Actigraphy data

Circadian rest-activity was measured by actigraphy. Each participant received an actigraph on the screening day visit. Due to limited data storage and battery life of each device, actigraphs were replaced on day eight and week four visits. Five participants received an extra actigraph because of a prolonged run-in phase. Although these actigraphs were replaced two to three times, their version was consistent within and across participants, and thus the impact of replacing these devices on the recording should be minimal. Participants were asked to continuously wear these devices on their non-dominant wrist until the end of the randomised phase.

GENEActiv software (ActivInsights Ltd., UK) was used to extract raw data from the GENEActiv actigraph. Raw data were corrected for gravity and summarised as a signal magnitude vector (SVM) using the following equation:

$$SVM=\sum |\sqrt{x^{2}\;+\;y^{2}\;+\;z^{2}}\;-\;g|$$

#### Data availability

Participants started recording a maximum of three weeks before randomisation (day −21), and the latest end date was 6 weeks post-randomisation (day 42). The earliest starting day of the Bayesian filter was set as day −14 (start of week −2) and truncated at day 28 (end of week 4) because a number of participants stopped collecting data after week 4 (Supplementary Figure S1).

#### Data preprocessing

Circadian rest-activity patterns were characterised from *daily* actigraphy data by non-parametric analysis using the R package GGIR package (version 1.11-0).^18^ Actigraphy non-wear time was detected by a default function of the GGIR algorithm. If more than 3 h of non-wear time were detected for a single day, the data of this day would be excluded for further analysis.

The non-parametric analysis does not assume that the circadian rest-activity patterns behave like sinusoidal waves,^19^ rendering it suitable for the analysis of circadian rest-activity patterns in patient groups who may have disrupted and less sinusoidal circadian rhythm. It has the additional advantage of preserving data on a day-to-day basis that facilitates the calculation of variability. The analysis generates two variables, M10 and L5. M10 corresponds to total activity in the *most active ten-hour period*, a proxy for daytime activity. L5 represents total activity in the *least active five-hour period*, a proxy for night-time activity or nocturnal arousal.^19^ Thus, higher M10 means elevated activity in the daytime, and higher L5, indicative of greater motor activity during sleep, is related to disturbed sleep. The onset time of M10 and L5 were also derived from the analysis. M10 onset time denotes the onset time of the most active ten-hour period of the day. L5 onset time corresponds to the onset time of the least active five-hour period of the day. We focused our analysis on the amount of M10 and its onset time.

Relative amplitude is calculated based on M10 and L5 amount as: (M10 − L5)/(M10 + L5). We also looked at the interdaily stability (IS) and intradaily variability (IV).

$$IS=\frac{\sum_{h=1}^{24}{\left(\bar{x_{h}}\;-\;\bar{x}\right)}^{2}}{\sum_{i=1}^{N}{\left(x_{i}\;-\;\bar{x}\right)}^{2}}$$

$$IV=\frac{\sum_{i=2}^{N}{\left(x_{i}\;-\;x_{i\;-\;1}\right)}^{2}/\left(N\;-\;1\right)}{\sum_{i=1}^{N}{\left(x_{i}\;-\;\bar{x}\right)}^{2}/N}$$

Where x is an observation, h is the time (in hours) of day, and N is the total number of datapoints in the data. Because the calculation of IS and IV requires data from multiple days, we computed IS and IV for each individual for each of the following period: run-in week, week 1, week 2, week 3, and week 4.

### The Bayesian filter

The Bayesian filter is a computational model of time series variability that seeks to tease apart different sources of variability from continuous observations.^14^ It was originally devised for affective variability, whereas in this study, the Bayesian filter is applied to daily actigraphy data. This model assumes that variability of a time series has two possible sources; one source is the random fluctuations of the observations as the time series varies around a mean, which is usually referred to as noise; the other source is a persistent shift of the time series due to a change of the underlying distribution, often called volatility.^13^ In the case of the variability of circadian rest-activity, noise could refer to the day-to-day natural fluctuations in activity level and active time, while volatility denotes a systematic shift in activity level and circadian phase that is unpredicted by the previous time series.

The Bayesian Filter was created by inverting a generative model of time series variability; the model inversion and parameter estimation process has been described previously.^14^ This generative model formulates that the observed value $y_{t}$ is drawn from a Gaussian distribution with mean ${mu}_{t}$ and standard deviation ${SD}_{t}$; the mean of the Gaussian distribution can change overtime at a rate controlled by a volatility parameter ${vmu}_{t}$. Volatility and noise in turn can change over time, at a rate controlled by two higher-level parameters kmu and vSD, respectively. By inverting this generative model, the Bayesian filter updates its belief about the distribution of parameter values at each time point sequentially using each new observation $y_{t}$. The parameter estimates of mean, volatility and noise are then calculated as the expected value of the parameter given the marginalised posterior distribution.

Because the Bayesian model assumes that the observed value lies in an unbounded space, while the mood or activity data is a positive number with a limited range, the observations were first scaled to values between 0.1 and 0.9, and then logit transformed. This way, the positive raw values were transformed into an unbounded space, while avoiding positive and negative infinity.

$$transformed\;score=logit\left(\frac{rawscore-\min}{\max-\min}*0.8+0.1\right)$$

The Bayesian model estimates the distribution of each of the five parameters at each time point. Apart from mu, which is estimated in normal space, all parameters were estimated in log space due to their non-negative property. One advantage of this model is its handling of missing data: if a time point is missing, the model systematically decreases the certainty of its estimates, meaning that it can provide parameter estimates even for time points with missing data.

**Fig. 1:**
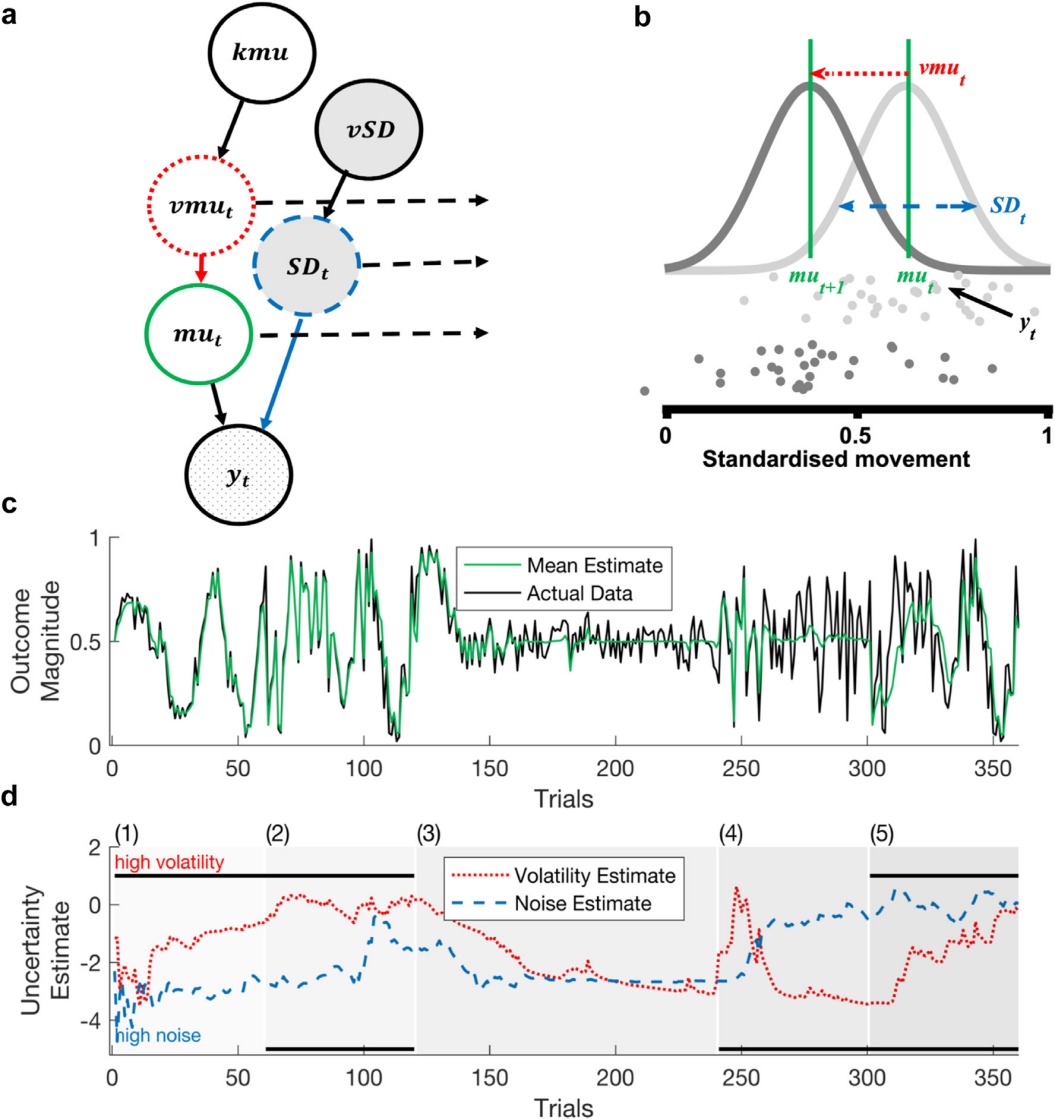
**A Bayesian filter was used to infer the different sources of variability.** (a) The design of the generative model. (b) An illustration of the relationship between volatility (vmu), noise (SD), and mean (mu). (c) Simulated data (black line) and estimated mean (green line) from different levels of volatility and noise. Segment (1) is when volatility is high and noise is low; segment (2) and (5) are when both volatility and noise are high; segment (3) is when both volatility and noise are low; segment (4) is when volatility is low while noise is high. These combinations of activity variability may have different clinical implications. For example, low volatility and high noise of daytime activity (4) may be related to psychomotor agitation, while low volatility and low noise (3) may indicate some form of psychomotor retardation commonly seen in depression. High volatility and low noise (1, 5) could be related to changes in, for example, changes in the external environment. (d) Estimated volatility (red dotted line) and noise (blue dashed line) from simulated data in (c). Adapted with permission from Pulcu et al.^14^

Using computational modelling, we estimated these two sources of variability for each type of observations, updating the parameter estimates in a hierarchical manner with each incoming observation (Fig. 1a and b). Even if an observation is missing, the model is able to provide parameter estimates, although with systematically decreased certainty. Fig. 1c and d gives an example of the relationship between observed data, estimated mean, volatility, and noise. The Bayesian filter was applied to each participants' data, for PA and NA, M10 and L5, activity level and activity onset time separately using MATLAB (version 2020b) (The MathWorks Inc., 2020).

### Ethics

Written informed consent was obtained from all participants prior to their inclusion in the study. The study protocol was registered on 22 January 2015 under the trial registration number ISRCTN91624955 (available at: https://www.isrctn.com/ISRCTN91624955). The study protocol was approved on 14 April 2015 by the National Research Ethics Service Committee South Central – Oxford A (reference number 15/SC/0109) and on 10 July 2015 by Oxford Health NHS Foundation Trust.

### Statistics

Participants were randomised by a central web-based computer algorithm that minimised separately on two variables related to prognosis: age (≤25 or >25 years) and sex (male or female). All individuals involved in trial recruitment and assessment visits, including patients, clinicians, and researchers were blinded to allocation. Central randomisation ensured complete allocation concealment. The computer-generated randomisation algorithm was designed by the software development team based at the University of Oxford's Department of Psychiatry.

The target sample size of 40 participants (20 per group) is calculated to provide over 90% power for detecting group differences in the primary outcome, mood.

Linear mixed models with fixed slopes and random intercepts for each participant and each data type were conducted in RStudio (version 2022.12.0+353) using the R package *lme4*^20^ and *lmerTest*.^21^ We examined correlations between two variables across time using *lmer*, accounting for both fixed effects and random effects that vary across participants. The acquired standardised regression coefficients *β* are a proxy for Pearson's correlation. Four linear mixed models were constructed for activity level, activity variability, activity onset time, and onset time variability, respectively, each with time as a repeated measure variable, and with within-subject intercept for each activity type (M10 or L5). M10 and L5 levels were included in the same linear mixed model because both M10 and L5 are both measures of activity level, only at different times of day. Volatility and noise were included in the same linear mixed model because both are measures of variability over time. Considering the timescale of lithium's action, the data were stratified into these following phases: run-in period (day −14 to day 0), week 1 (day 1–7), week 2 (day 8–14), week 3 (day 15–21), and week 4 (day 22–28), and these five stages were used as repeated measure variables in the linear mixed models. Binning data into weeks to render a week-level estimate rather than estimating day-level effect is primarily based on the timing of lithium's action. Because we are interested in the effect of lithium above and beyond its impact on PA and its volatility as previously reported,^14^ each model controlled for log-transformed PA and the volatility of PA, in addition to age and sex.

For M10 and L5 activity level, a mixed linear model was built with one parameter *activity_type* indicating the type of activity (M10 or L5): Activity_level [continuous] ∼ group_assignment [lithium or placebo] ∗ activity_type [M10 or L5] ∗ phase [run-in, week 1, week 2, week 3, week 4] + log_PA [continuous]+ PA_volatility [continuous] + Age [continuous] + Sex [F, M] + Season_of_intake [Spring, Summer, Autumn, Winter] + (1 + activity_type | participant).

For the variability of M10 and L5 levels derived from the Bayesian filter, a linear mixed model was constructed with one factor *activity_type* indicating the type of activity (M10 or L5), and another factor *variability_type* indicating the type of variability (volatility or noise). This way, the volatility and noise of M10 and L5 could be separately estimated by releveling the model to different contrast levels. Supplementary analyses were conducted controlling for whether the recording day is a weekday or weekend and controlling for this additional covariate did not alter the findings.

Similarly, we constructed the following linear mixed model fitted to relative amplitude, and weekly IS and IV: Relative_amplitude/IS/IV ∼ group_assignment ∗ phase + log_positive_affect + volatility_positive_affect + Age + Sex + Season + (1|ID).

In all models above, the timing variable was referenced to the pre-randomisation baseline, and the group variable was referenced to the placebo group. We focused on the interaction effect between phase and lithium treatment relative to the placebo group and the pre-randomisation baseline, for M10 level (activity model) and M10 volatility (variability model) respectively. Although L5, L5 volatility, and the noise of M10 and L5 were not the main outcome of interest these levels were included in the linear mixed models to improve the model estimation of individual-specific intercepts. We adopted a significance level of α = 0.05 for all statistical analyses, but we do note that we did not correct for multiple comparisons for the four post-randomisation weeks, and the interpretation of week-specific effects should therefore be cautioned. We also tested the effect of lithium on relative amplitude, interdaily stability, and intradaily variability, which are measures of circadian rhythmicity. All linear mixed models were fitted through Restricted Maximum Likelihood and converged successfully. All analyses are based on intent-to-treat, meaning that we analysed participants based on their initial assignments.

### Role of funders

The funders had no role in the study design, data collection, data analysis, interpretation of the results, or the writing of the manuscript.

## Results

**Fig. 2:**
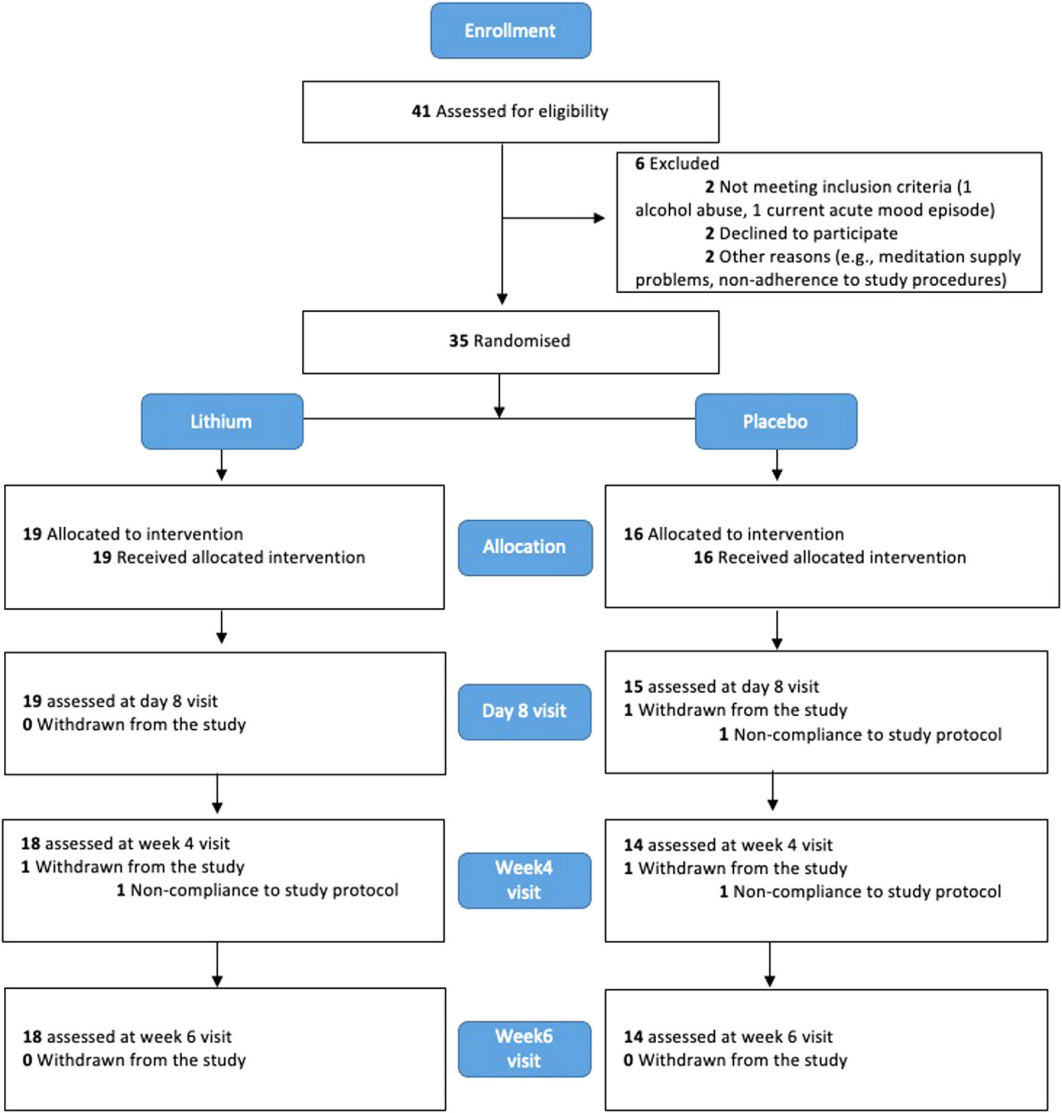
**CONSORT flow diagram**.

From August 2015 to Jan 2018, 41 participants were screened for eligibility and six were excluded. Thirty-five participants were randomised and initiated treatment; 19 received lithium and the other 16 received placebo. Of the 19 participants randomised to lithium, 18 (95%) completed the study. Of the 16 participants randomised to placebo, 14 (88%) completed the study. No participants experienced an acute episode during the follow-up period that would have led to their exclusion from the study (Fig. 2).

Table 4 showed the demographic information in the lithium group and placebo group respectively (See Supplementary Table S1 for demographic information by sex). Most of the recruited participants (77%) were diagnosed with bipolar II disorder. See Supplementary Figure S2 for lithium serum level and Supplementary Table S2 for self-reported adherence.

**Table 4:** Demographic details of study participants.

	Randomised treatment	
	Lithium (N = 19)	Placebo (N = 16)
**Sex: n (%)**		
Male	8 (42%)	7 (44%)
Female	11 (58%)	9 (56%)
**Ethnicity: n (%)**		
Asian	0	1
Black	1	0
Hispanic	1	0
Mixed	1	1
Other	1	1
White British	13	9
White other	1	1
Not reported	1	3
**Age (years): mean (SD)**	28.37 (9.86)	34.69 (13.86)
**BMI: mean (SD)**	25.41 (6.28)	26.49 (4.95)
**Subjective affect: mean (SD)**		
Positive affect	10.71 (3.50)	10.44 (3.01)
Negative affect	8.71 (3.25)	10.68 (4.18)
**ASRM total scores: mean (SD)**	2.83 (3.81)	3.63 (3.93)
**QIDS total scores: mean (SD)**	9.56 (6.25)	11.25 (4.95)
**Missing days: median**	13.5	17.0
**Recording days: median**	50	50
**Bipolar disorder subtype: n (%)**		
BD I	3 (16%)	4 (25%)
BD II	16 (84%)	11 (69%)
BD NOS	0 (0%)	1 (6%)
**Seasons of intake: n (%)**		
Spring	3 (16%)	3 (19%)
Summer	3 (16%)	5 (31%)
Autumn	4 (21%)	4 (25%)
Winter	9 (47%)	4 (25%)
**Actigraphy metrics: mean (SD)**		
M10 level - absolute unit	56.61 (18.24)	58.10 (19.57)
L5 level - absolute unit	3.89 (0.76)	3.96 (0.58)
M10 onset time - hours after 0 am	12.47 (1.98)	12.12 (2.49)
L5 onset time - hours after 0 am	25.34 (1.90)	25.61 (1.93)

Abbreviations: BD, bipolar disorder; BMI, body mass index; NOS, not otherwise specified.

### Cross-sectional association between mood, activity level, and activity onset time

Cross-sectionally and within-individual, M10 and L5 activity levels were not correlated with each other (standardised correlation coefficient *β* = 0.047, p = 0.214, linear mixed model, ibid.). M10 and L5 activity onset time were positively correlated, *β* = 0.308, p < 0.001, such that later daytime activity onset time is associated with later night-time rest onset time. M10 level was positively associated with log-transformed positive affect (*β* = 0.150, p = 0.003), but had no association with log-transformed negative affect (*β* = −0.061, p = 0.114), meaning that higher daytime activity level was selectively associated with higher PA had no relation with NA. M10 onset time was not associated with either log-transformed PA (*β* = −0.007, p = 0.870) or NA (*β* = 0.019, p = 0.579). The volatility of M10 was positively associated with the volatility of M10 onset time (*β* = 0.593, p < 0.001), such that more volatile daytime activity level is associated with more volatile onset time of daytime activity. Neither the volatility of M10 (*β* = 0.143, p = 0.069) nor the volatility of M10 onset time (*β* = 0.036, p = 0.711) was associated with the volatility of PA (Supplementary Figure S3).

### Lithium reduces daytime activity level while specifically increasing volatility of activity

**Fig. 3:**
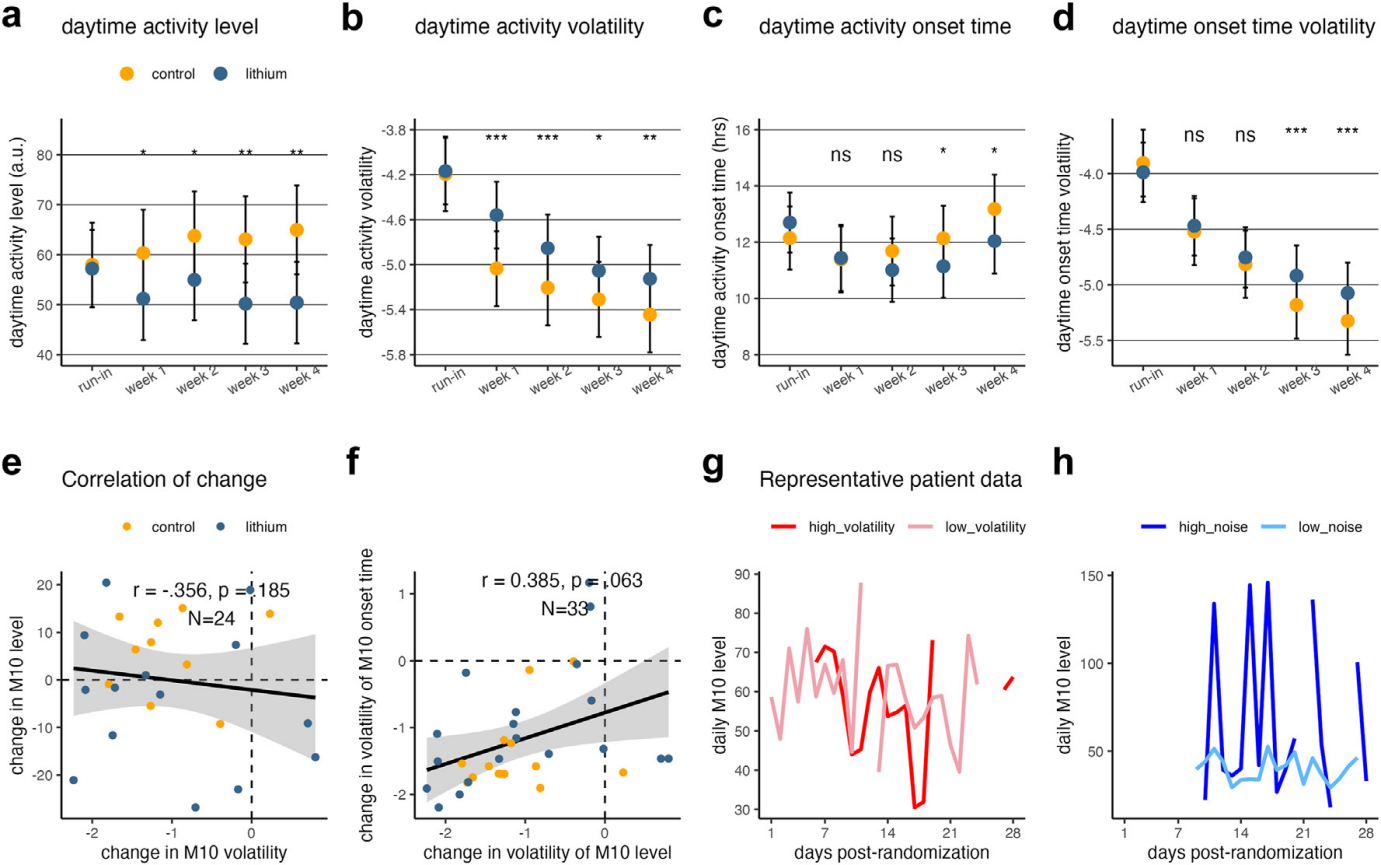
**Results of the mixed linear model, for M10 level (top panel) and M10 onset time (middle panel) respectively (N = 34)**. (a) Daytime activity level as a function of week and group assignment. (b) Daytime activity volatility as a function of time and group assignment. (c) Daytime activity onset time as a function of week and group assignment. (d) Daytime activity onset time volatility as a function of week and group assignment. (e) The change in M10 level from pre-randomisation baseline to week 4 was not associated with the change in the change in the volatility of M10 level. (f) The change in volatility of M10 level from pre-randomisation baseline to week 4 was marginally significantly associated with the change in volatility of the M10 onset time (p = 0.06, linear mixed model). Panel F has more data points than panel E because the Bayesian filter still renders variability estimates when missing data is present. (g, h) Representative actigraphy data from a participant with higher versus low volatility (g) and high versus low noise (h). Note. The error bars in a–d indicate the 95% confidence interval; importantly, the overlap of the 95% CI of the two groups does not necessarily imply non-significance. The asterisks indicate significance level: n.s., non-significant; ∗p < 0.05, ∗∗p < 0.01; ∗∗∗p < 0.001.

Results from the linear mixed models show that after randomisation, lithium significantly decreased M10 by 18.8% compared to placebo group at baseline in week 1 (unstandardised regression coefficient B = −8.368, t = −2.134, unadjusted p = 0.033, Cohen's d = −0.129, linear mixed model, ibid.), as well as in week 2 (18.1% reduction from baseline, B = −8.048, t = −2.023, unadjusted p = 0.043, Cohen's d = −0.122), week 3 (27.2% reduction from baseline, B = −12.124, t = −3.146, unadjusted p = 0.002, Cohen's d = −0.189), and week 4 (30.9% reduction from baseline, B = −13.761, t = −3.282, unadjusted p = 0.001, Cohen's d = −0.196) (Fig. 3a) (Supplementary Table S3). Supplementary Figure S4 summarises the effect of lithium on PA and its volatility for comparison. For the variability parameters derived from the Bayesian Filter, lithium increased M10 volatility relative to placebo in week 1 (B = 0.450, t = 5.017, p < 0.001, Cohen's d = 0.173), week 2 (B = 0.326, t = 3.530, p < 0.001, Cohen's d = 0.122), week 3 (B = 0.229, t = 2.431, p = 0.015, Cohen's d = 0.084), and week 4 (B = 0.292, t = 3.054, p = 0.002, Cohen's d = 0.105) (Fig. 3b). M10 volatility decreased significantly from baseline in both placebo group (B = −1.251, t = −18.346, p < 0.001) and lithium group (B = −0.958, t = −14.304, p < 0.001) by week 4. Lithium was also found to increase L5 volatility in week 2, week 3, and week 4, but since it did not have a significant effect on L5 level itself, these results were less interpretable (Supplementary Table S4). Finally, lithium significantly increased interdaily stability by week 4 relative to placebo group at baseline, while showing no impact on relative amplitude or intradaily variability (Supplementary Figure S5).

### Lithium advances daytime activity onset time and increases volatility of onset time

Compared to the placebo group, the lithium group had an M10 onset time 1.55 h earlier than that of the placebo group at baseline in week 3 (B = −1.550, t = −2.354, p = 0.019, Cohen's d = −0.142, linear mixed model, *ibid.*) and 1.69 h earlier in week 4 (B = −1.691, t = −2.372, p = 0.018, Cohen's d = −0.143), but not in week 1 (B = −0.506, t = −0.749, p = 0.455, Cohen's d = −0.045) or week 2 (B = −1.231, t = −1.805, p = 0.071, Cohen's d = −0.109) (Fig. 3c) (Supplementary Table S5).

For the variability parameters, the lithium group's M10 onset time volatility was significantly higher relative to the placebo group at baseline in week 3 (B = 0.345, t = 4.103, p < 0.001, Cohen's d = 0.141) and week 4 (B = 0.333, t = 3.898, p < 0.001, Cohen's d = 0.134), but not in week 1 (B = 0.136, t = 1.701, p = 0.089, Cohen's d = 0.059) and week 2 (B = 0.145, t = 1.753, p = 0.080, Cohen's d = 0.060) (Fig. 3d). M10 onset time volatility significantly decreased from baseline to week 4 in both placebo group (B = −1.420, t = −23.304, p < 0.001) and lithium group (B = −1.086, t = −18.145, p < 0.001). Lithium also increased the volatility of L5 onset time in week 1 and week 2, but this result is difficult to interpret due to the absence of a direct effect on L5 onset time (Supplementary Table S6).

Finally, we examined the correlation among changes across different domains. The reduction in M10 level from baseline to week 4 was not significantly correlated with the increase in M10 volatility, controlling for age, sex and season of intake (Pearson's *r* = −0.356, t = −1.382, p = 0.185) (Fig. 3e). Similarly, the advancing of M10 onset time from baseline to week 4 was not significantly correlated with the increase in the volatility of M10 onset time, controlling for age, sex and season (Pearson's *r* = −0.078, t = −0.334, p = 0.742). The amount of change in M10 onset time was not correlated with the amount of change in M10 activity level, and the amount of change in M10 onset time or activity level was not correlated with the amount of change in PA, either for lithium group alone or for all patients combined (Supplementary Figure S6). Finally, the increase in M10 volatility from baseline to week 4 was marginally significantly correlated with the increase in the volatility of M10 onset time across patients (Pearson's *r* = 0.385, t = 1.938, p = 0.063) (Fig. 3f). Fig. 3g and h showed representative patients with higher versus low volatility (g) and noise (h), respectively.

## Discussion

The present study used a real-time high frequency monitoring approach via digital devices to investigate the causal effects of lithium on circadian rest-activity in patients with BD under a randomised, placebo-controlled design.^12^^,^^15^ We found that lithium treatment reduced daytime activity (M10, most active 10 h) level and advanced the onset time of M10. Further, we used a Bayesian filter previously shown to capture clinically relevant aspects of affective variability to differentiate different sources of activity variability, volatility and noise.^14^ We found that in addition to its direct effects on daytime activity level and onset time, lithium also increases the volatility of both daytime activity and its onset time, while not influencing any measures of noise.

This randomised placebo-controlled study examined the causal relationship between lithium and circadian rest-activity in patients with BD. Furthermore, this study unravels the chronological order of changes in activity and affective domains following lithium treatment and demonstrates that the earliest changes are in circadian rest-activity. We found that lithium had an early impact on circadian rest-activity independent of changes in affective symptoms, reducing daytime activity level while advancing the onset time of daytime activity. After controlling for daily measured affect, lithium's impact remains significant until the end of the fourth week. A previous study has noted that a correction of circadian disruption is essential for the early phases of lithium therapy and for the distinction between lithium responders and lithium non-responders.^22^ Furthermore, significant changes in circadian rest-activity triggered by lithium occurred as early as the first week after taking lithium, which is earlier than when the first significant change in PA occurred.^14^ Although this observation is not formally tested and should be interpreted with caution, we conjecture that there may be a mechanism by which mood is regulated by an earlier behavioural effect. Although we cannot prove that with the present results (a mediation analysis would require a much larger study), the results of our study are consistent with this hypothesis.

By identifying early and independent changes in circadian rest-activity, we can also shed light on how to develop early biomarkers of lithium's action. The ACE model characterises BD as a combination of symptoms across three domains: Activity, Cognition, and Emotion.^1^ A further understanding of the chronology of changes within the activity and affective domains following lithium may suggest that circadian rest-activity metrics can potentially serve as an early biomarker for mood stabilisation post-treatment. Currently, psychiatrists who treat patients with BD rely on a ‘trial-and-error’ approach that requires patients to take lithium for a number of months in order to determine the degree of response.^23^ The stabilisation of circadian rest-activity after lithium treatment may provide early indications of the effectiveness of lithium, which can help to identify the most appropriate mood stabiliser for each patient. Future studies will require longer-term follow-up to link early-stage changes with long-term clinical effectiveness.

Previous studies have found that lithium can modulate the phase of circadian rhythms, though the findings are mixed. For example, studies in healthy subjects and in rats have reported a phase-delay effect of lithium.^10^ In contrast, another study involving patients with BD found that lithium advanced the phase of temperature rhythms.^24^ Our previous systematic review revealed a potential association between lithium and greater morningness based on several observational studies.^12^ In another study, lithium-responsive patients with BD showed higher levels of morningness than lithium non-responders.^25^ Federoff and McCarthy reported a bipolar I disorder patient with disruption of circadian rhythms; an advanced phase was noted after taking low-dose lithium, changing from very low to high morningness in this patient.^26^

In the current study, lithium was found to advance the onset time of most active 10 h (a proxy for daytime activity onset time) in patients with BD in week 3 and 4 compared with the placebo, yet had no effect on the onset time of the least active 5 h (a proxy for sleep onset time). This may suggest that lithium has a phase-advancing effect in particular for the timing of daytime activity. Our findings are supported by a recent prospective observational study which found that circadian phase disturbances as measured by wearable devices precede mood symptoms.^27^ This raises the possibility that the phase-advancing effect of lithium might mediate the mood-stabilising effect, although this would require a much larger clinical study with prolonged treatment to test this hypothesis.

Our experimental study design revealed that lithium decreased circadian rest-activity compared to the placebo over the four weeks following randomisation. One possible explanation for this activity-decreasing effect may be the sedating effect of lithium or its side effects such as fatigue and drowsiness. However, there have also been previous studies in animal models of BD that support the hypothesis that lithium might stabilise circadian activity by reducing elevated activity level. Various methods have been employed to trigger increased activity, mimicking manic-like behaviour, such as paradoxical sleep deprivation,^28^ stimulants,^29^ constant light,^30^ and mutations in the clock genes.^31^ All four studies found that lithium decreased elevated activity levels, which is consistent with our findings. Relatedly, we observed that lithium significantly increased interdaily stability of circadian rest-activity by week 4. Supporting this, a recent cross-sectional study reported that lithium monotherapy was associated with higher stability across days.^32^ Taken together, these results suggest that the observed decrease in daytime activity may reflect a stabilising effect of lithium, potentially indicating clinical improvement, rather than being merely a side effect of lithium treatment.

In our study, we observed that increased volatility may represent an independent aspect of lithium's mechanism of action. The previously mentioned changes—activity reduction and circadian phase advancing—did not correlate with changes in their respective volatilities, potentially due to lack of power. The changes in M10 volatility were marginally significantly correlated with alterations in the volatility of M10 onset time, potentially suggesting the presence of a common mechanism through which lithium exerts its effects. Volatility decreased from baseline to week 4 in both groups, likely reflecting the Bayesian filter's parameter estimation becoming more refined over time rather than a true reduction in M10 variability.

In this context, we interpret increased volatility not as a sign of instability, but as an indicator of flexibility. Increased volatility suggests that the lithium group's activity level and onset time became more patterned, where an initial change becomes more likely to be maintained rather than to rapidly dissipate. This change suggests a potentially beneficial early effect that may tip a patient towards recovery, consistent with a recent proposal on the mechanism of BD rooted in dynamical systems theory.^33^ In this view, patients with BD could be conceptualised as trapped in a vicious cycle of mood instability.^34^^,^^35^ Lithium initially acts as a catalyst in causing perturbations that help propel patients from a trapped state toward a healthier one, which is manifested as increased volatility after perturbation. It would be expected that the volatility would decrease after reaching a new equilibrium, but this hypothesis requires a much longer monitoring time and is beyond the scope of this study. Additionally, other abovementioned mechanisms of lithium, such as activity reduction and phase advancing, may contribute to a healthier resilience. These mechanisms may improve synchronisation with the light-dark cycle and social rhythms. Such alignment can be crucial in stabilising mood symptoms and reinforcing a healthier mental state. For example, a delayed circadian phase has been linked to depressive episodes,^36^ and the phase-advancing effect of lithium may be one of the underlying mechanisms in its treatment.

While this study provides valuable insight into the mechanism of action of lithium, the following limitations should be acknowledged. First of all, the study was designed to investigate the early effects of lithium. As such, there was no prolonged follow-up period to determine whether these early changes in circadian rest-activity had any clinical implications. Furthermore, given the short timescale, it was not possible to distinguish between lithium responders (Li-R) and non-responders (Li-NR) among the participants. It has been found that Li-R and Li-NR exhibit different circadian rhythm phenotypes^25^; as a result, this heterogeneity may obscure the differences in circadian rest-activity between lithium and placebo groups. This study also had a small sample size, and included a subgroup of patients with non-severe BD experiencing significant mood instability. It may not be appropriate to generalise the results to all patients with BD. Also, actigraphy provides an indirect measure of circadian function. Specifically, actigraphy cannot capture core biological circadian markers, such as melatonin or core body temperature rhythms.^37^ Additionally, actigraphy can be influenced by external movements, such as regular clinical visits in Oxlith, which may mask underlying circadian patterns. Future studies could leverage emerging computational tools that measure circadian rhythm using ambient lighting and sleep stage scoring to triangulate the estimation of circadian rhythm.^27^^,^^38^, ^39^, ^40^ Finally, this study provided observational but not direct experimental evidence of the chronological order of lithium's impact. Future studies can test out whether direct intervention on activity (e.g., restricting high-impact activity during the day, encouraging variety of activity and affective experience) can lead to the same mood and circadian rhythm benefits.

Our study implemented an experimental medicine design, encompassing high-frequency data acquisition of activity and affect, along with computational modelling, to examine the effects of lithium on circadian rest-activity. This randomised, placebo-controlled study delineated a causal relationship between lithium and alterations in circadian rest-activity among patients with BD. Our findings suggest a potentially causal effect of lithium on stabilising, and advancing the phase of, daytime activity. Furthermore, using a computational model, we discovered that lithium increases volatility of circadian rest-activity, a phenomenon interpreted as increased flexibility. Additionally, significant changes in circadian rest-activity metrics were observed as early as one week after commencing lithium therapy, suggesting their potential utility as biomarkers for lithium responsiveness. These results imply the potential role of circadian rest-activity in mediating the mood stabilisation effects of lithium. Future research, employing a larger sample size and prolonged monitoring period, is necessary to further explore these findings.

## Contributors

NX: Conceptualisation, Data Curation, Formal Analysis, Investigation, Methodology, Visualisation, Writing – Original Draft Preparation; YY: Conceptualisation, Formal Analysis, Investigation, Methodology, Visualisation, Writing – Original Draft Preparation, Visualisation; KS and JG: Data Curation, Funding Acquisition, Investigation, Project Administration, Resources, Supervision, Writing – Review & Editing; MB: Data Curation, Investigation, Methodology, Resources, Supervision, Writing – Review & Editing.

NX, YY, and MB have accessed and verified the data; NX, YY, and MB were responsible for the decision to submit the manuscript. All authors have read and approved the final version of this manuscript.

## Data sharing statement

The study did not obtain consent to upload data onto open platforms; however, de-identified actigraphy data can be made available upon reasonable request to KS (kate.saunders@psych.ox.ac.uk) for research groups that have ethical approval in place on a by-project basis. Analysis code related to this paper is available at https://github.com/ryanyim98/oxlith_volatilitynoise.

## Declaration of interests

NX, YY, and JG declare no conflict of interests. KS receives support from Oxford Health NIHR Biomedical Research Centre. KS has also received grant funding from Boehringer Ingelheim and non-financial support from Big Health in the form of no cost access to the digital sleep improvement programme, Sleepio, for use in clinical research, outside the submitted work. MB receives grants from Wellcome Trust. MB has acted as a consultant for Janssen Research, P1vital Ltd, Boehringer and CHDR, and Engrail Therapeutics, and was previously a paid employee of P1vital Ltd.
