# Supplementary material for: Effect of lithium on circadian activity level and flexibility in patients with bipolar disorder: results from the Oxford Lithium Trial
Source: eBioMedicine. 2025 Apr 2;115:105676. doi: 10.1016/j.ebiom.2025.105676 (PMC11999483; doi:10.1016/j.ebiom.2025.105676)
Supplement: Supplementary Figures S1–S6 [file mmc1.docx]

**Supplementary Information for manuscript:**

Effect of Lithium on Circadian Activity Level and Flexibility in Patients with Bipolar Disorder: Results from The Oxford Lithium Trial


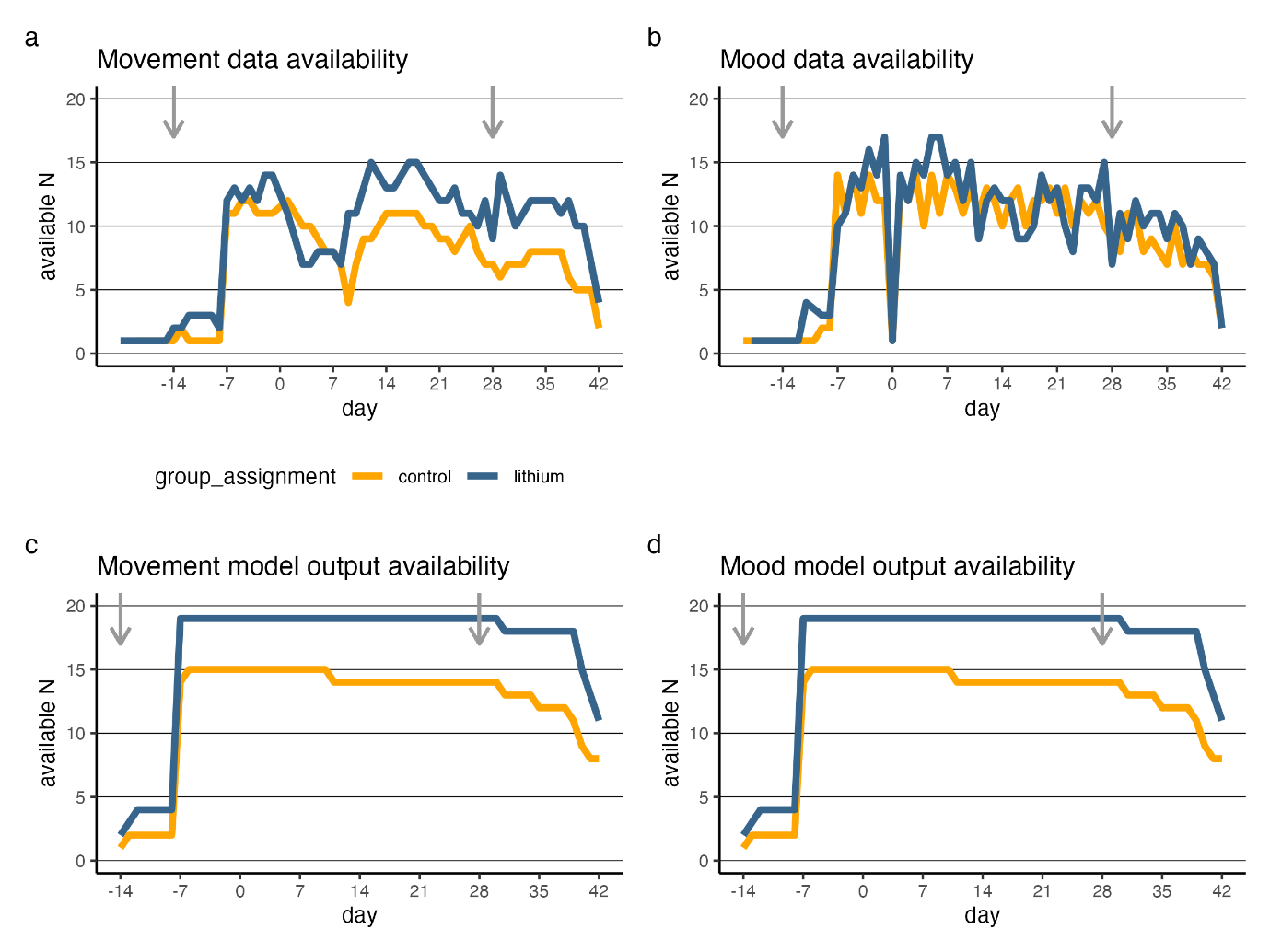


**Figure S1.** Availability of activity data, mood data, activity model parameters, and mood model parameters across the study. The grey arrows show the time window used in this study, -14 to 28 days.

**Figure S2.** Lithium serum level reported at each visit. Each black point represents the lithium serum level of each participant. The red dotted line denotes the minimal lithium serum level (0.4 mmol/L) of our targeted lithium therapeutic range (0.4-1.0 mmol/L).


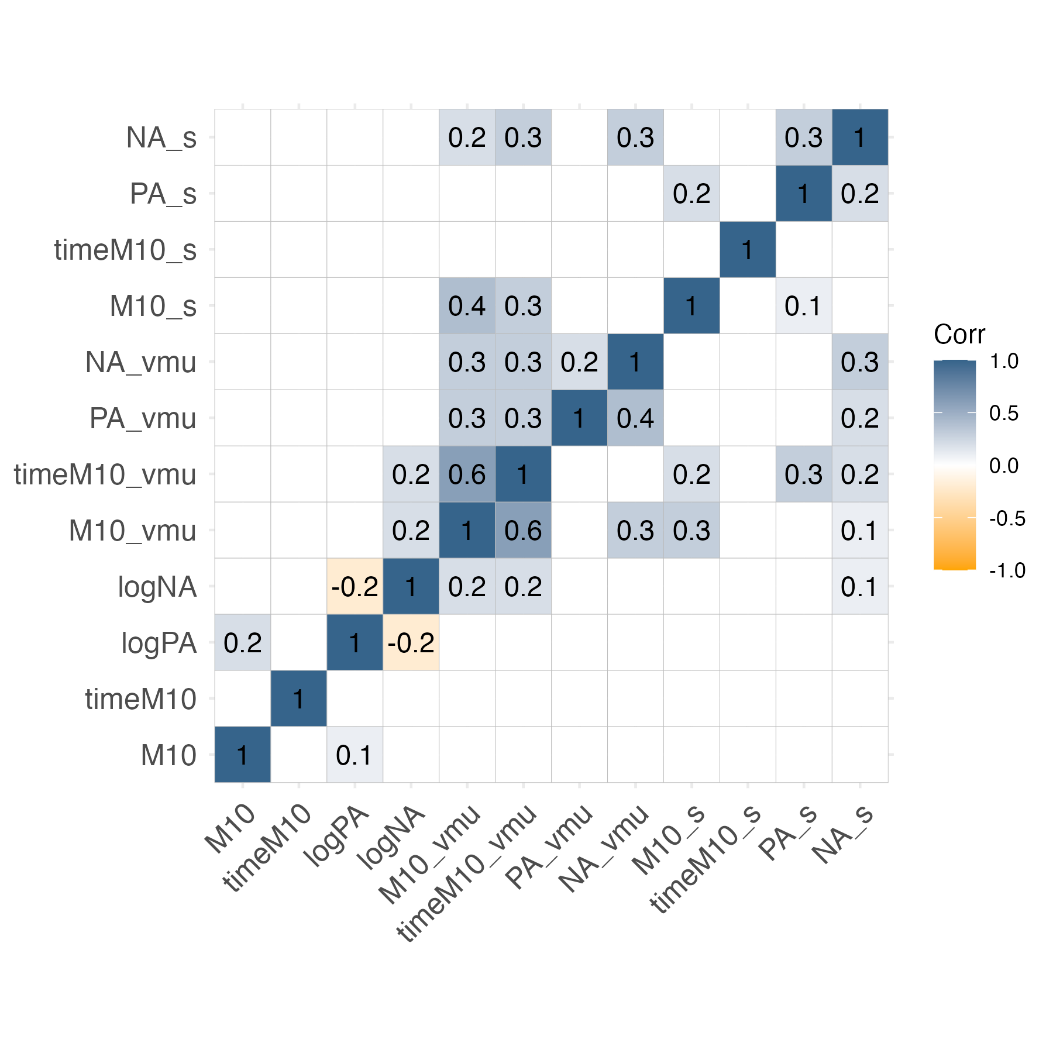


**Figure S3.** The intercorrelations among activity level, activity onset time, and mood, and their respective model estimates. Model formula: variable 1 ~ variable 2 + (1|subject). All daily data before and after randomisation were used. Abbreviations: PA, positive affect; NA, negative affect; vmu, volatility; s, noise. Blank squares indicate non-significant findings (p ≥ .05).


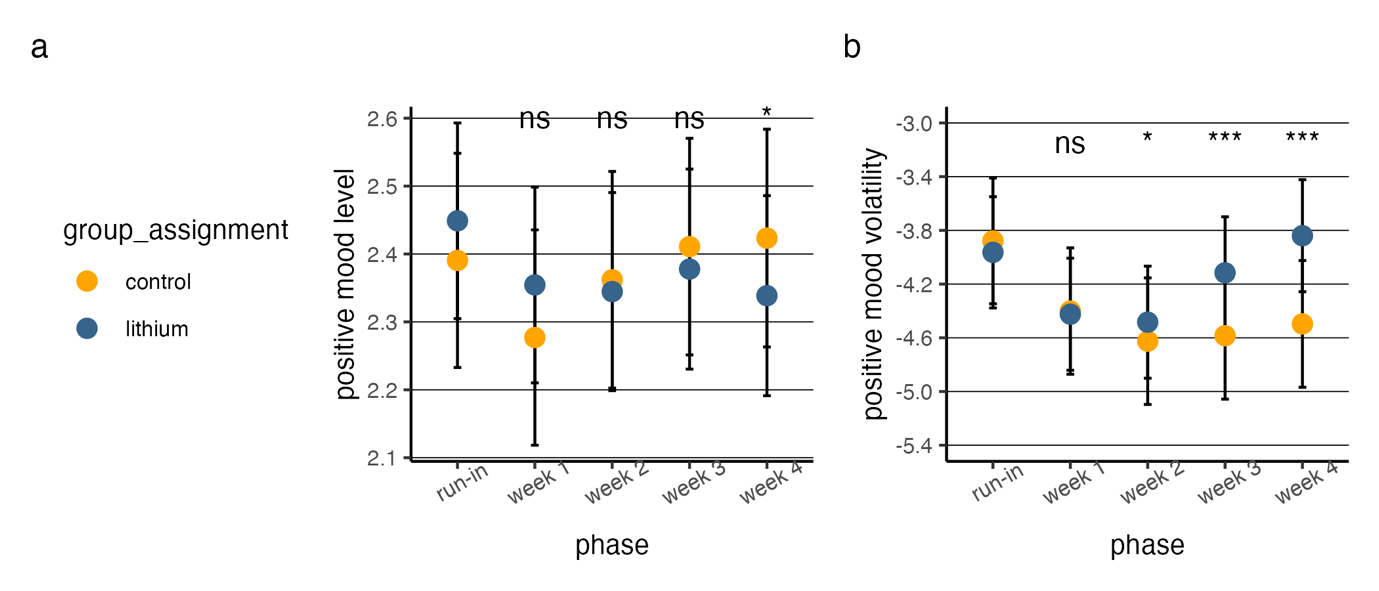


**Figure S4.** Effect of lithium on log-transformed positive affect (PA) (A) and volatility of PA (B). The PA difference between lithium group and placebo group was only observed in week 4 post-randomization, and the difference in PA volatility between two groups were significant at week 2, 3 and 4. Model formula: PA_rating ~ Allocation * phase + Age + Sex + Season + (1| participant) for (A) and PA_variability ~ Allocation * variability_type * phase + Age + Sex + Season + (variability_type| participant) for (B).

**Effect of lithium on relative amplitude, interdaily stability and intradaily variability**

**Results.**

As shown by the figure below, we found that relative to baseline, lithium increased interdaily stability in week 4 (B = 0.189, t = 2.713, p = .008) relative to placebo group at baseline, while IS decreased significantly for the placebo group by week 4 (B = -0.160, t = -3.134, p = .002). We did not find an effect of lithium on intradaily variability (p > .052, linear mixed model). Relative amplitude was not altered across four weeks of treatment (p > .260, linear mixed model).

Finally, across two groups, the volatility of M10 level, but not the noise, was positively associated with IS cross-sectionally in each week (standardised beta = 0.261, t = 3.014, p = .003), but their amount of change from baseline to week 4 was not significantly correlated (Pearson’s r = 0.400, p = .118, N = 26).


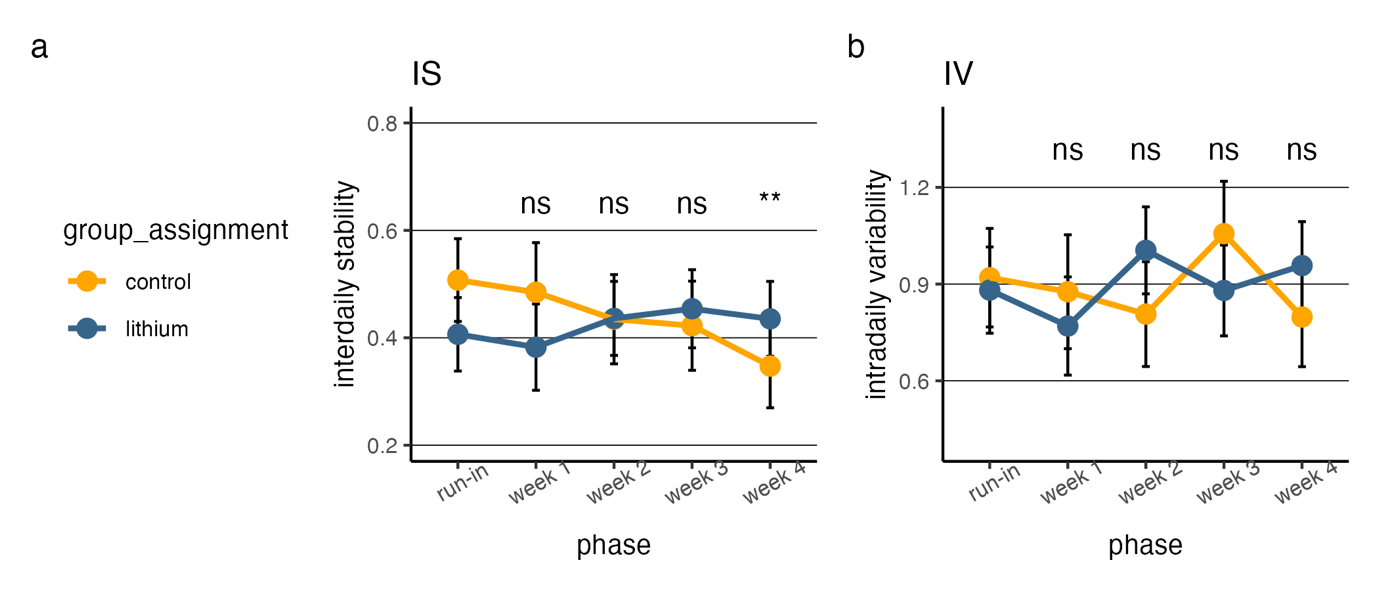


**Figure S5**. Effect of lithium on interdaily stability and intradaily variability

**
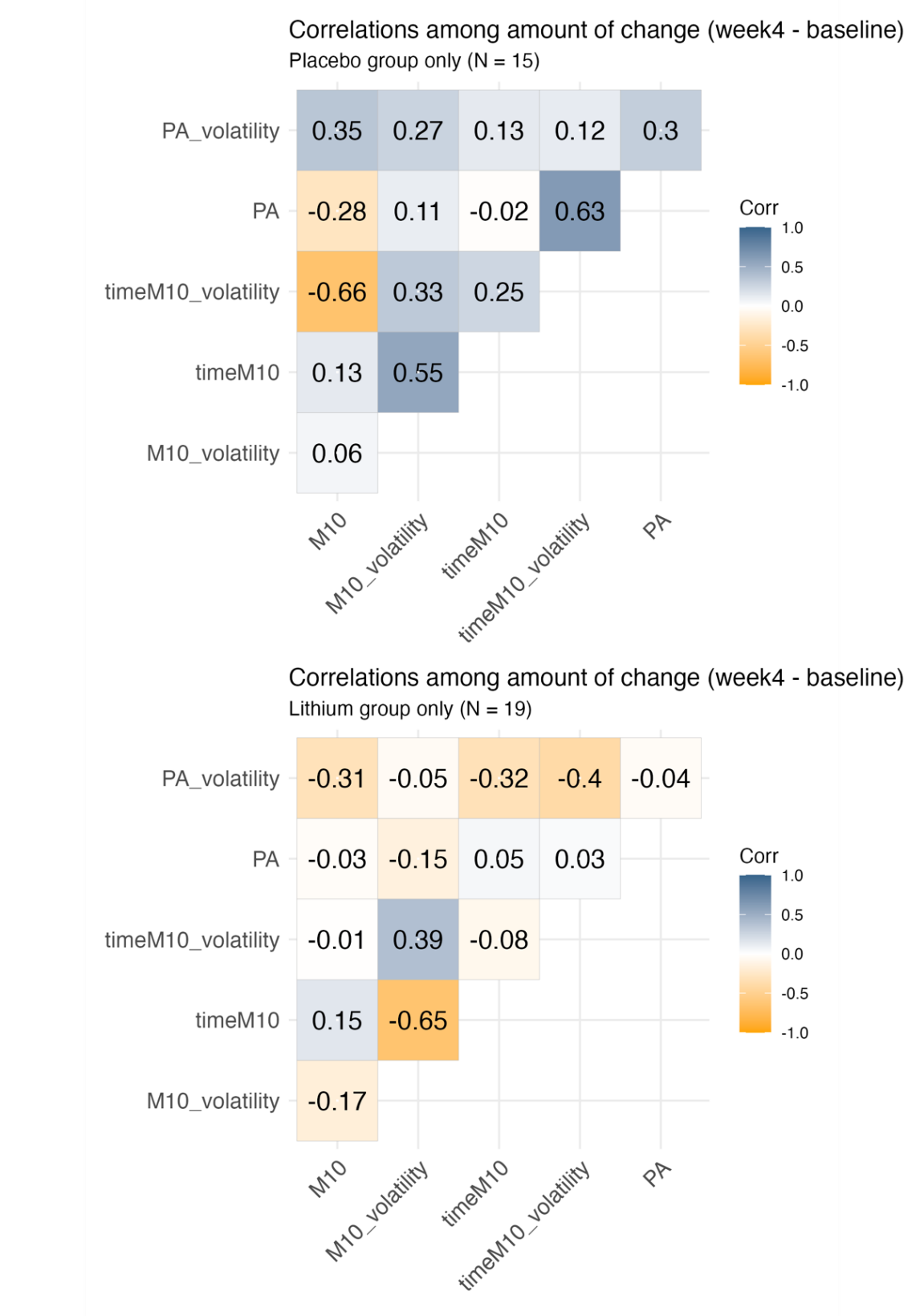
**

**Figure S6.** Correlations in the change (calculated as week 4 - baseline) of positive affect, M10, M10 onset time, and their volatility, for all patients (top panel) or for lithium group only (bottom panel). The numbers denote correlation coefficients from simple Pearson correlations; the crosses denote non-significance (p ≥ .05).
