# Supplementary material for: Effect of lithium on circadian activity level and flexibility in patients with bipolar disorder: results from the Oxford Lithium Trial
Source: eBioMedicine. 2025 Apr 2;115:105676. doi: 10.1016/j.ebiom.2025.105676 (PMC11999483; doi:10.1016/j.ebiom.2025.105676)
Supplement: Supplementary Tables S1–S6 [file mmc2.docx]

**Supplementary Information for manuscript:**

Effect of Lithium on Circadian Activity Level and Flexibility in Patients with Bipolar Disorder: Results from The Oxford Lithium Trial

|  | **Randomised treatment** | | | |
| --- | --- | --- | --- | --- |
|  | Lithium (N=19) | | Placebo (N=16) | |
|  | Male (N=8) | Female (N=11) | Male (N=7) | Female (N=9) |
| **Ethnicity** | | | |  |
| Asian | 0 | 0 | 0 | 1 |
| Black | 0 | 1 | 0 | 0 |
| Hispanic | 0 | 1 | 0 | 0 |
| Mixed | 0 | 1 | 0 | 1 |
| Other | 1 | 0 | 1 | 0 |
| White British | 5 | 8 | 5 | 4 |
| White Other | 1 | 0 | 1 | 0 |
| Not reported | 1 | 0 | 0 | 3 |
| **Age (years): mean (SD)** | | | | |
|  | 27.00 (5.50) | 29.36 (12.29) | 34.00 (15.64) | 35.22 (13.27) |
| **BMI: mean (SD)** | | | |  |
|  | 22.70 (2.05) | 27.37 (7.61) | 26.61 (4.23) | 26.40 (5.71) |
| **Subjective affect: mean (SD)** | | | | |
| Positive affect | 8.71 (3.59) | 12.17 (2.73) | 8.88 (2.31) | 11.48 (3.09) |
| Negative affect | 7.78 (3.07) | 9.38 (3.35) | 8.87 (3.37) | 11.89 (4.40) |
| **Missing days: median** | | | | |
|  | 9.0 | 21.5 | 15.5 | 17.0 |
| **Recording days: median** | | | | |
|  | 50 | 50 | 48 | 50 |
| **Bipolar disorder subtype: n (%)** | | | | |
| BD I | 0 | 3 | 2 | 2 |
| BD II | 8 | 8 | 4 | 7 |
| BD NOS | 0 | 0 | 1 | 0 |
|  |  |  |  |  |
| **Seasons of intake: n (%)** | | | | |
| Spring | 2 | 1 | 3 | 3 |
| Summer | 1 | 2 | 0 | 3 |
| Autumn | 1 | 3 | 2 | 1 |
| Winter | 4 | 5 | 2 | 2 |
| **Actigraphy metrics: Mean (SD)** | | | | |
| M10 level - absolute unit | 60.73 (24.88) | 52.96 (9.58) | 62.74 (26.62) | 55.52 (15.71) |
| L5 level - absolute unit | 4.21 (0.92) | 3.61 (0.46) | 3.97 (0.47) | 3.95 (0.66) |
| M10 onset time - hours after 0am | 12.64 (2.05) | 12.36 (2.03) | 12.59 (2.01) | 11.83 (2.79) |
| L5 onset time - hours after 0am | 25.14 (2.19) | 25.52 (1.72) | 25.49 (1.45) | 25.67 (2.24) |
| Abbreviations: BD, bipolar disorder; BMI, body mass index; NOS, not otherwise specified. | | | | |

**Table S1.** Demographic information by Sex.

| Reported adherence | Day 4 visit | Day 8 visit | Week 4 visit | Week 6 visit |
| --- | --- | --- | --- | --- |
| More than 65%: n (%) | 19 (100%) | 19 (100%) | 18 (95%) | 17 (94%) |
| Between 50-65%: n (%) | 0 | 0 | 1 (5%) | 1 (6%) |
| Between 25-49%: n (%) | 0 | 0 | 0 | 0 |
| Less than 25%: n (%) | 0 | 0 | 0 | 0 |

**Table S2.** Reported adherence to lithium treatment through the trial period

|  | **Estimate** | **Std. Error** | **df** | **t value** | **p** |
| --- | --- | --- | --- | --- | --- |
| **M10 activity** | | | | | |
| lithium:phase (post week1) | -8.368 | 3.922 | 1103.932 | -2.134 | **0.033** |
| lithium:phase (post week2) | -8.048 | 3.978 | 1103.924 | -2.023 | **0.043** |
| lithium:phase (post week3) | -12.124 | 3.854 | 1108.657 | -3.146 | **0.002** |
| lithium:phase (post week4) | -13.761 | 4.193 | 1116.670 | -3.282 | **0.001** |
| **L5 activity** | | | | | |
| lithium:phase (post week1) | -0.534 | 3.844 | 1093.391 | -0.139 | 0.889 |
| lithium:phase (post week2) | 0.174 | 3.864 | 1093.520 | 0.045 | 0.964 |
| lithium:phase (post week3) | 0.640 | 3.702 | 1093.762 | 0.173 | 0.863 |
| lithium:phase (post week4) | 1.161 | 3.941 | 1093.130 | 0.278 | 0.781 |
| **Covariates** | | | | | |
| logPA | 4.531 | 1.467 | 726.335 | 3.089 | **0.002** |
| PA_volatility | -0.617 | 0.458 | 780.998 | -1.348 | 0.178 |
| Age | -0.010 | 0.058 | 209.937 | -0.170 | 0.865 |
| Sex: Male | 1.604 | 1.390 | 238.376 | 1.154 | 0.250 |
| Season: Spring | 0.086 | 2.106 | 212.853 | 0.041 | 0.967 |
| Season: Summer | -0.359 | 1.810 | 205.515 | -0.198 | 0.843 |
| Season: Winter | -0.529 | 1.844 | 210.697 | -0.281 | 0.779 |

**Table S3.** Mixed linear model examining the effect of Lithium on activity level. Model formula: activity _level ~ Allocation * activity _type * phase + logPA + PA_volatility + Age + Sex + Season + (1+ activity _type | ID), where activity _type is either M10 or L5, and phase is run-in period to week 4.

|  | **Estimate** | **Std. Error** | **df** | **t value** | **p** |
| --- | --- | --- | --- | --- | --- |
| **M10 volatility** | | | | | |
| lithium:phase (post week1) | 0.450 | 0.090 | 3374.077 | 5.017 | **< .001** |
| lithium:phase (post week2) | 0.326 | 0.092 | 3375.279 | 3.530 | **< .001** |
| lithium:phase (post week3) | 0.229 | 0.094 | 3377.346 | 2.431 | **0.015** |
| lithium:phase (post week4) | 0.292 | 0.096 | 3379.237 | 3.054 | **0.002** |
| **M10 noise** | | | | | |
| lithium:phase (post week1) | 0.135 | 0.090 | 3379.759 | 1.509 | 0.131 |
| lithium:phase (post week2) | 0.161 | 0.092 | 3382.678 | 1.745 | 0.081 |
| lithium:phase (post week3) | -0.036 | 0.094 | 3386.063 | -0.385 | 0.701 |
| lithium:phase (post week4) | -0.082 | 0.096 | 3389.612 | -0.863 | 0.388 |
| **L5 volatility** | | | | | |
| lithium:phase (post week1) | 0.164 | 0.090 | 3378.020 | 1.829 | 0.068 |
| lithium:phase (post week2) | 0.421 | 0.092 | 3380.152 | 4.561 | **< .001** |
| lithium:phase (post week3) | 0.330 | 0.094 | 3383.495 | 3.507 | **< .001** |
| lithium:phase (post week4) | 0.501 | 0.096 | 3386.880 | 5.244 | **< .001** |
| **L5 noise** | | | | | |
| lithium:phase (post week1) | 0.070 | 0.090 | 3383.476 | 0.787 | 0.431 |
| lithium:phase (post week2) | 0.085 | 0.092 | 3387.106 | 0.925 | 0.355 |
| lithium:phase (post week3) | 0.143 | 0.094 | 3391.046 | 1.516 | 0.130 |
| lithium:phase (post week4) | 0.088 | 0.096 | 3395.102 | 0.918 | 0.359 |
|  |  | **Covariates** |  |  |  |
| logPA | 0.097 | 0.028 | 3086.096 | 3.429 | **0.001** |
| PA_volatility | 0.028 | 0.009 | 2953.722 | 3.015 | **0.003** |
| Age | 0.000 | 0.003 | 26.047 | 0.053 | 0.958 |
| Sex: Make | 0.127 | 0.077 | 27.356 | 1.657 | 0.109 |
| Season: Spring | -0.023 | 0.120 | 27.092 | -0.190 | 0.850 |
| Season: Summer | -0.029 | 0.111 | 26.229 | -0.259 | 0.797 |
| Season: Winter | -0.058 | 0.108 | 26.737 | -0.539 | 0.594 |

**Table S4.** Mixed linear model examining the effect of Lithium on activity variability. Model formula: activity_variability ~ Allocation * variability_type * activity_type * phase + logPA + PA_volatility + Age + Sex + Season + (1 + variability_type * activity_type| participant), where activity _type is either M10 or L5, variability_type is either volatility or noise, and phase is run-in period to week 4.

|  | **Estimate** | **Std. Error** | **df** | **t value** | **p** |
| --- | --- | --- | --- | --- | --- |
| **M10 onset time** | | | | | |
| lithium:phase (post week1) | -0.505 | 0.675 | 1095.270 | -0.749 | 0.454 |
| lithium:phase (post week2) | -1.231 | 0.682 | 1097.252 | -1.805 | 0.071 |
| lithium:phase (post week3) | -1.550 | 0.658 | 1098.040 | -2.354 | **0.019** |
| lithium:phase (post week4) | -1.693 | 0.714 | 1097.752 | -2.372 | **0.018** |
| **L5 onset time** | | | | | |
| lithium:phase (post week1) | 0.441 | 0.673 | 1096.544 | 0.655 | 0.513 |
| lithium:phase (post week2) | 0.347 | 0.680 | 1096.532 | 0.510 | 0.610 |
| lithium:phase (post week3) | 0.376 | 0.655 | 1092.696 | 0.575 | 0.566 |
| lithium:phase (post week4) | -0.165 | 0.708 | 1081.078 | -0.222 | 0.816 |
| **Covariates** | | | | | |
| logPA | -0.468 | 0.308 | 995.912 | -1.519 | 0.129 |
| PA_volatility | 0.033 | 0.094 | 1011.185 | 0.353 | 0.724 |
| Age | -0.041 | 0.026 | 27.365 | -1.555 | 0.131 |
| Sex: Male | 0.069 | 0.597 | 28.828 | 0.115 | 0.909 |
| Season: Spring | 0.663 | 0.924 | 28.052 | 0.717 | 0.479 |
| Season: Summer | 0.261 | 0.851 | 26.619 | 0.307 | 0.761 |
| Season: Winter | 0.403 | 0.833 | 27.749 | 0.484 | 0.632 |

**Table S5.** Mixed linear model examining the effect of Lithium on M10 and L5 activity onset time. Model formula: activity_onset_time ~ Allocation * time_type * phase + logPA + PA_volatility + Age + Sex +Season + (1 + time_type| participant), where time_type is either L5 onset time or M10 onset time, and phase is run-in period to week 4.

|  | **Estimate** | **Std. Error** | **df** | **t value** | **p** |
| --- | --- | --- | --- | --- | --- |
| **M10 onset time volatility** | | | | | |
| lithium:phase (post week1) | 0.136 | 0.080 | 3373.904 | 1.701 | 0.089 |
| lithium:phase (post week2) | 0.145 | 0.083 | 3375.575 | 1.753 | 0.080 |
| lithium:phase (post week3) | 0.345 | 0.084 | 3378.297 | 4.103 | **<.001** |
| lithium:phase (post week4) | 0.333 | 0.086 | 3381.717 | 3.898 | **<.001** |
| **M10 onset time noise** | | | | | |
| lithium:phase (post week1) | -0.034 | 0.080 | 3382.857 | -0.431 | 0.667 |
| lithium:phase (post week2) | -0.092 | 0.082 | 3386.714 | -1.112 | 0.266 |
| lithium:phase (post week3) | -0.112 | 0.084 | 3390.783 | -1.332 | 0.183 |
| lithium:phase (post week4) | -0.047 | 0.085 | 3394.670 | -0.553 | 0.580 |
| **L5 onset time volatility** | | | | | |
| lithium:phase (post week1) | 0.205 | 0.080 | 3374.232 | 2.560 | **0.011** |
| lithium:phase (post week2) | 0.373 | 0.083 | 3375.877 | 4.522 | **<.001** |
| lithium:phase (post week3) | 0.029 | 0.084 | 3378.888 | 0.347 | 0.729 |
| lithium:phase (post week4) | 0.073 | 0.086 | 3381.550 | 0.855 | 0.393 |
| **L5 onset time noise** | | | | | |
| lithium:phase (post week1) | 0.001 | 0.080 | 3380.959 | 0.016 | 0.987 |
| lithium:phase (post week2) | -0.120 | 0.082 | 3384.346 | -1.460 | 0.144 |
| lithium:phase (post week3) | -0.051 | 0.084 | 3388.616 | -0.613 | 0.540 |
| lithium:phase (post week4) | -0.039 | 0.085 | 3392.830 | -0.454 | 0.650 |
|  |  | Covariates |  |  |  |
| logPA | 0.069 | 0.025 | 2738.510 | 2.738 | 0.006 |
| PA_volatility | 0.001 | 0.008 | 2516.683 | 0.160 | 0.873 |
| Age | -0.005 | 0.003 | 26.516 | -1.926 | 0.065 |
| Sex: Male | 0.020 | 0.057 | 28.255 | 0.345 | 0.733 |
| Season: Spring | 0.046 | 0.088 | 27.770 | 0.520 | 0.607 |
| Season: Summer | -0.008 | 0.082 | 26.737 | -0.102 | 0.920 |
| Season: Winter | 0.022 | 0.079 | 27.335 | 0.276 | 0.785 |

**Table S6.** Mixed linear model examining the effect of Lithium on activity onset time’s variability. Model formula: onset time variability ~ Allocation * variability_type * time_type * phase + logPA + PA_volatility + Age + Sex + Season + (1 + variability_type * time_type| participant), where time _type is either M10 onset time or L5 onset time, variability_type is either volatility or noise, and phase is run-in period to week 4.
